# Perioperative Cerebral Protection and Monitoring of Acute Stanford Type A Aortic Dissection: A Retrospective Cohort Study

**DOI:** 10.3390/jcdd13010012

**Published:** 2025-12-24

**Authors:** Yi Jiang, Jianing Wang, Chang Liu, Yong Liu, Lin Mi, Tian Fang, Yongqing Cheng, Hoshun Chong, Dongjin Wang, Yunxing Xue

**Affiliations:** 1 Department of Thoracic and Cardiovascular Surgery, Nanjing Drum Tower Hospital, Graduate School of Peking Union Medical College, Chinese Academy of Medical Science & Peking Union Medical College, Peking Union Medical College Graduate School, Nanjing 210008, China; 2 Department of Thoracic and Cardiovascular Surgery, Nanjing Drum Tower Hospital, The Affiliated Hospital of Nanjing University Medical School, Nanjing 210008, China; 3 Institute of Cardiothoracic Vascular Disease, Nanjing University, Nanjing 210008, China

**Keywords:** type A aortic dissection, deep hypothermic circulatory arrest, cerebral protection, NIRS

## Abstract

Background: Optimal cerebral protection strategies for acute Stanford type A aortic dissection (aTAAD) surgery remain controversial. This study aimed to evaluate the role of near-infrared spectroscopy (NIRS)-guided monitoring and its association with clinical outcomes. Methods: We retrospectively analyzed 619 patients undergoing aTAAD surgery (Hemi-Arch, Total-Arch, or Arch-Stent procedures). Intraoperative cerebral oxygenation was monitored using NIRS, with the magnitude of desaturation quantified as ΔNIRS. We assessed correlations between ΔNIRS and nasopharyngeal temperature, employed generalized additive models (GAM) to analyze nonlinear relationships with major adverse cardiovascular events (MACE), and used piecewise logistic regression to identify procedure-specific ΔNIRS risk thresholds. Results: ΔNIRS showed a significant positive correlation with lower temperatures in Total-Arch (R = 0.486, *p* < 0.001) and Arch-Stent (R = 0.216, *p* < 0.001) groups. GAM analysis revealed a nonlinear, accelerating relationship between higher ΔNIRS and increased log odds of MACE in Hemi-Arch and Total-Arch groups. Procedure-specific ΔNIRS thresholds were identified: 8.5% for Hemi-Arch, 19.6% for Total-Arch, and 20.9% for Arch-Stent. Patients with ΔNIRS above these thresholds had significantly higher rates of stroke and MACE. Conclusions: This study identifies ΔNIRS as a significant, procedure-dependent intraoperative monitoring indicator in aTAAD surgery, and the proposed risk thresholds provide a rationale for real-time NIRS-guided clinical decision-making.

## 1. Introduction

Acute Type A aortic dissection (aTAAD) represents a life-threatening cardiovascular emergency characterized by substantial morbidity and mortality [1]. The urgency of aTAAD necessitates immediate medical intervention, with surgical repair remaining the definitive treatment [2].

However, a critical component of arch surgery involves the use of deep hypothermic circulatory arrest (DHCA) to facilitate a bloodless field, yet this technique carries a significant risk of cerebral ischemia and neurological injury [3]. Since the 1970s, various perioperative cerebral protection strategies have been implemented during DHCA, with hypothermia and cerebral perfusion emerging as the most widely adopted and effective approaches [4,5]. Hypothermia reduces cerebral metabolic demands across temperature gradients, while cerebral perfusion maintains cerebral blood flow. Current clinical practices employ three principal cerebral perfusion modalities: unilateral selective antegrade cerebral perfusion (uSACP), bilateral cerebral perfusion (BCP), and retrograde cerebral perfusion (RCP) [6,7,8]. While each technique has distinct advantages and limitations, considerable controversy persists regarding the optimal temperature, underscoring the urgent requirement for reliable real-time cerebral function assessment methods.

Cerebral near-infrared spectroscopy (NIRS) serves as the predominant clinical modality for monitoring cerebral ischemia–hypoxia through rapid assessment of cerebral metabolic status [9,10]. Cerebral NIRS quantifies regional oxygen saturation via differential absorption of hemoglobin species (600–900 nm), providing real-time perfusion/oxygenation feedback. Current criteria for abnormal NIRS during DHCA include > 20% reduction from baseline or absolute values < 50%. Despite limited predictive value for stroke or neurocognitive dysfunction compared to cumbersome alternatives, NIRS remains widely adopted [11,12,13]. This study mainly investigates real-time regional oxygen saturation (rSO_2_) measurements detected by NIRS [14].

This study assesses individualized cerebral protection strategies during DHCA across a range of aTAAD procedures. We leverage dynamic NIRS variation as a novel metric to link intraoperative perfusion data to surgical outcomes, aiming to guide patient-specific strategy selection based on arch pathology and projected arrest duration.

## 2. Methods

### 2.1. Subject

**Figure 1:**
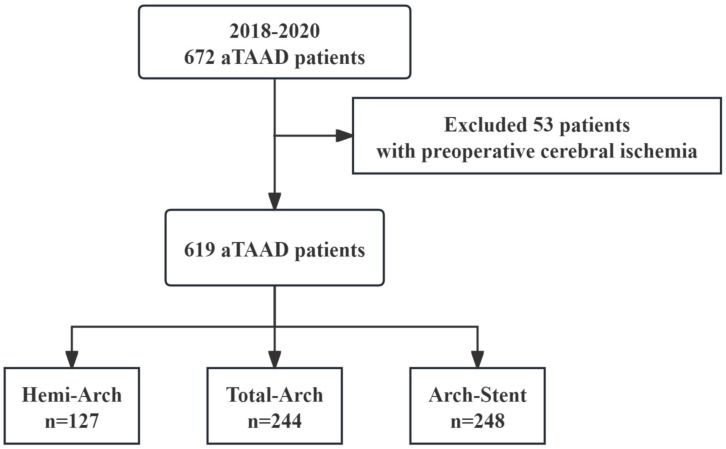
Flow chart of patients enrolled in this study. aTAAD, acute Type A aortic Dissection.

From January 2018 to December 2020, our center performed circulation-stopping surgery on 672 patients with acute Stanford Type A aortic dissection. The previous study assessed preoperative cerebral ischemia as a strong risk factor for postoperative cerebral complications; therefore, 53 patients were excluded due to preoperative cerebral ischemia. All patients were diagnosed by enhanced CT or transthoracic echocardiography (TTE) before surgery, and were transferred to cardiothoracic intensive care unit (CICU) to improve preoperative preparation and emergency surgical treatment (Figure 1).

Ethical clearance for this study was granted by the Medical Ethics Committee of Nanjing Drum Tower Hospital (approval number: 2020-185-01), and it adhered to the ethical guidelines outlined in the Declaration of Helsinki for medical research involving human subjects. Prior to their enrollment, all patients received informed consent over the phone.

### 2.2. Surgical Procedure

The surgical method of the arch determines the expected cycle stop time, so different hypothermia ranges and cerebral perfusion strategies will be selected according to the surgical method of the arch [15]. Surgical method of the arch and specific surgical operations are shown in Supplementary File S1.

#### 2.2.1. Hemi-Arch Replacement

Indication: ① No intimal rupture in the aortic arch; ② the superior branch of the aortic arch was not involved or mildly involved, and the aortic arch was slightly detached; ③ no obvious expansion of the aortic arch (diameter < 40 mm); ④ the patient could not tolerate long-term cardiopulmonary bypass due to age and poor preoperative basic condition.

Operation steps: The aorta was transected distal to the affected part of the aortic arch or the planned replacement part, and the artificial blood vessel was anastomosed to the aortic arch end to end.

#### 2.2.2. Total Arch Replacement

Indication: ① Aortic arch intima tear and aortic arch structure severely damaged; ② Aortic arch dilation ≥ 45 mm;

Operation steps: ① Total arch replacement with a quad-branched artificial vessel, accompanied by frozen elephant trunk stent implantation in the descending aorta (minimally invasive, Shanghai); ② modified island anastomosis in the aortic arch cavity using frozen elephant trunk stent.

#### 2.2.3. Arch Stent Group

Indication: ① No break on the large curved side of the arch; ② no expansion of the arch (diameter < 40 mm); ③ no Marfan syndrome or other connective tissue diseases; Operation steps: The ascending aorta was transected 2 cm before the opening of the trunk of the head and arm, and the fenestrating stent was placed into the aortic arch and the true lumen of the descending aorta. The fenestrating point was aligned with the opening of the branch vessel of the aortic arch, and the stent was released anteriorly. After the release, it was confirmed that the branch vessel opening of the aortic arch was located at the opening window of the stent, and the artificial blood vessel was anastomosed with the proximal end of the stent end to end [16].

### 2.3. Intraoperative Cerebral Protection and Monitoring

Intraoperative brain perfusion methods include antegrade cerebral perfusion (ACP), retrograde cerebral perfusion (RCP) and no cerebral perfusion while deep hypothermic circulatory arrest (DHCA). Intraoperative cerebral oxygen monitoring was used to measure the effectiveness of cerebral perfusion using near-infrared reflectance spectroscopy (NIRS). NIRS is for bilateral monitoring, using the Nonin sen smart x-100 device. When this value is less than 20% of the baseline or less than 50% of the absolute value, we consider cerebral perfusion inadequate, and additional cerebral perfusion measures need to be taken, including increasing cerebral perfusion methods and cerebral perfusion flow [17]. ACP was performed via axillary artery or innominate artery/common carotid artery. The initial perfusion flow was 3–5 mL/kg/min, and the flow rate is calculated based on the body surface area multiplied by 2.2 to 2.8. It was adjusted according to NIRS. RCP is retrograde perfusion via superior vena cava, and the monitoring of venous pressure < 25 mmHg [18]. During the operation, according to the method of arch operation, the estimated time of stopping circulation and whether there is cerebral perfusion, different low temperature intervals of stopping circulation were selected: <20 °C, 20–24 °C and ≥24 °C.

### 2.4. Postoperative Management and Follow-Up

After the operation, the patient was transferred to CICU for monitoring and treatment. Neurological function is assessed every 6–8 h after entry. For patients with persistent coma and positive physical signs after surgery, further neurological imaging examination should be performed depending on the stability of the condition. Ischemic stroke, as defined by clinical presentation, physical examination, and imaging evidence. The rest shall be dealt with according to the condition.

### 2.5. Outcome Definitions

Stroke was defined as a new postoperative cerebral infarction confirmed by imaging.

Major Adverse Cardiovascular Events (MACE) were defined as a composite endpoint comprising: (1) cardiovascular death, (2) non-fatal myocardial infarction, and (3) non-fatal stroke [19].

### 2.6. Statistical Analysis

Statistical analysis used SPSS 23.0 and R 4.2.2. Continuous variables are presented as mean ± standard deviation or median [interquartile range] based on distribution normality, assessed by the Kolmogorov–Smirnov test. Categorical variables are presented as n (%). Group comparisons utilized ANOVA/*t*-test (normal continuous), Wilcoxon rank-sum test (non-normal continuous), or Fisher’s exact test (categorical). Correlations were assessed using Pearson’s or Spearman’s coefficients.

To evaluate the association between ΔNIRS and MACE, standard logistic regression was performed. We further explored potential nonlinearity using: (1) a logistic generalized additive model (GAM) with smooth terms [20], fitted via the mgcv package (version 1.8–42); and (2) a piecewise logistic regression model to identify threshold effects [21], fitted using the segmented package (version 2.1–4). Model comparisons were conducted using the log-likelihood ratio test. A two-sided *p* value < 0.05 indicated statistical significance.

## 3. Results

### 3.1. Patients Baseline Characteristics

This study included 619 patients with aTAAD and divided them into three groups according to the type of arch surgery (Figure 1): Hemi-Arch (*n* = 127), Total-Arch replacement (*n* = 244), and Arch-Stent (*n* = 248). Baseline characteristics differed significantly among groups (Table 1). Patients undergoing Hemi-Arch repair were older (median age: 58 years), while those in the Total-Arch group were youngest (median: 51 years; *p* < 0.001). The Total-Arch group also had a higher proportion of males. Although most comorbidities were comparable, a history of cerebral infarction was more frequent in the Hemi-Arch group (7.9% vs. 2.0% vs. 3.6%, *p* = 0.022). Further preoperative details, such as Marfan syndrome and diabetes are provided in Supplementary Table S1.

Surgical strategies varied accordingly. In the Hemi-Arch group, femoral cannulation alone was most common (51.2%), whereas combined femoral–axillary cannulation predominated in the Total-Arch group (73.4%). Due to the limited sample size, no subgroup analysis of arterial cannulation approaches was performed. Cerebral perfusion approaches also differed: DHCA alone and DHCA + ACP were used similarly in Hemi-Arch cases (50.4% vs. 41.7%), while ACP was dominant in Total-Arch (88.1%) and Arch-Stent (53.6%) procedures. RCP was infrequent overall. Moderate hypothermia (20–24 °C) was the preferred circulatory arrest temperature range across all groups.

Postoperative outcomes did not differ significantly. Stroke rates were 6.3%, 6.1%, and 7.7% in the Hemi-Arch, Total-Arch, and Arch-Stent groups, respectively (*p* = 0.650), with corresponding in-hospital mortality of 11.8%, 9.4%, and 8.9% (*p* = 0.777).

### 3.2. Results in Hemi-Arch Group

**Figure 2:**
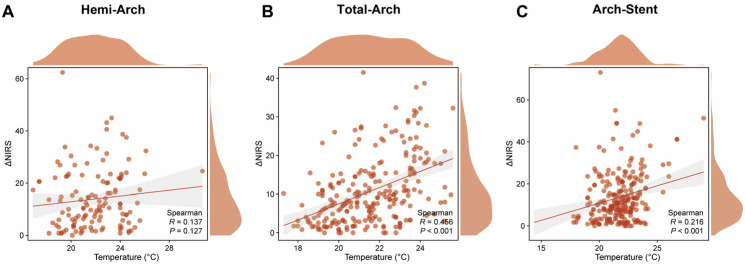
Correlation between the lowest nasopharyngeal temperature and ΔNIRS. Scatter plots with fitted lines display the relationship between intraoperative temperature and the magnitude of cerebral oxygen desaturation (ΔNIRS) for each surgical group. Statistical analysis was performed using Spearman’s rank correlation. (**A**) Hemi-Arch group: R = 0.137, *p* = 0.127. (**B**) Total-Arch group: R = 0.456, *p* < 0.001. (**C**) Arch-Stent group: R = 0.216, *p* < 0.001.

**Figure 3:**
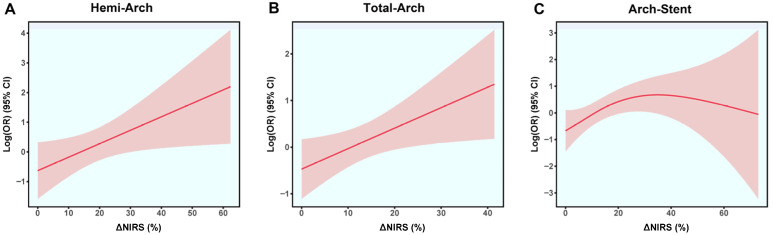
Nonlinear association between ΔNIRS and MACE analyzed by logistic generalized additive models (GAM). Smooth curves (solid lines) depict the relationship between the predictor (ΔNIRS) and the log odds ratio (log[OR]) for MACE across the three surgical groups, with shaded areas representing the 95% confidence intervals. (**A**) Hemi-Arch group, (**B**) Total-Arch group, and (**C**) Arch-Stent group.

**Figure 4:**
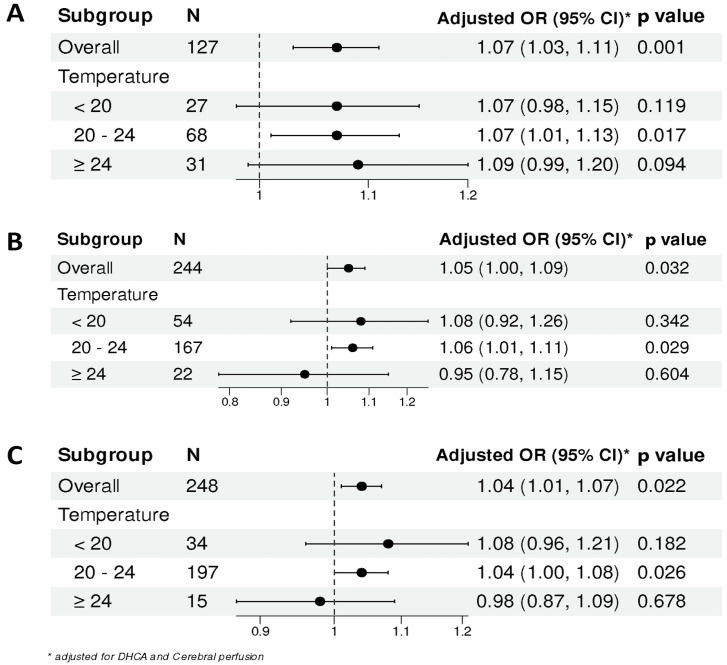
Forest plot of adjusted odds ratios for the association between ΔNIRS and MACE, stratified by circulatory arrest temperature. The analysis was performed separately for (**A**) Hemi-Arch group, (**B**) Total-Arch group, and (**C**) Arch-Stent group.

In the Hemi-Arch group, a weak positive correlation was observed between the lowest intraoperative temperature (Nasopharyngeal temperature) and ΔNIRS, which did not reach statistical significance (Spearman R = 0.137, *p* = 0.127; Figure 2A). Generalized Additive Model (GAM) analysis revealed a positive nonlinear association between ΔNIRS and the log odds of adverse outcomes (Figure 3A). The risk increased progressively with higher ΔNIRS values, showing a steeper rise without a clear plateau or inflection point. Using a piecewise logistic regression model, an optimal ΔNIRS cutoff of 8.5% was identified for this cohort (Supplementary Figure S1A and Table S2). Patients with ΔNIRS ≥ 8.5% (*n* = 77) had significantly higher rates of in-hospital mortality (16.9% vs. 4.0%, *p* = 0.028), stroke (10.4% vs. 0%, *p* = 0.022), and MACE (23.4% vs. 4.0%, *p* = 0.003) compared to the low ΔNIRS group (<8.5%), despite similar DHCA durations (*p* = 0.311; Table 2). Forest plot analysis, adjusted for DHCA and cerebral perfusion strategy, confirmed that higher ΔNIRS was significantly associated with increased risk in the overall group (OR = 1.07, 95% CI: 1.03–1.11, *p* = 0.001) and in the 20–24 °C subgroup (OR = 1.07, 95% CI: 1.01–1.13, *p* = 0.017; Figure 4A).

### 3.3. Results in Total-Arch Group

For patients undergoing Total-Arch replacement, a significant moderate positive correlation was found between temperature and ΔNIRS (Spearman R = 0.456, *p* < 0.001; Figure 2B). The GAM curve demonstrated a similarly positive and accelerating relationship between ΔNIRS and the risk of the outcome MACE (Figure 3B). Application of a piecewise logistic regression model determined an optimal ΔNIRS cutoff of 19.6% (Supplementary Figure S1B and Table S3). The high ΔNIRS group (≥19.6%) exhibited significantly higher stroke (17.9% vs. 3.9%, *p* = 0.004) and MACE (23.1% vs. 11.2%, *p* = 0.044) rates compared to the low ΔNIRS group (< 19.6%), with a borderline higher mortality (17.9% vs. 7.8%, *p* = 0.068; Table 3). Adjusted analysis indicated that ΔNIRS remained a significant predictor of adverse outcomes overall (OR = 1.05, 95% CI: 1.00–1.09, *p* = 0.032) and within the 20–24 °C subgroup (OR = 1.06, 95% CI: 1.01–1.11, *p* = 0.029; Figure 4B).

### 3.4. Results in Arch-Stent Group

In the Arch-Stent group, ΔNIRS was weakly but significantly correlated with lower temperatures (Spearman R = 0.216, *p* < 0.001; Figure 2C). The GAM plot revealed a distinct pattern: the log(OR) initially increased with ΔNIRS, peaked, and then showed a slight descending trend at very high ΔNIRS values, suggesting a potential threshold effect (Figure 3C). Piecewise logistic regression analysis identified an optimal ΔNIRS cutoff of 20.9% (Supplementary Figure S1C and Table S4). Patients with ΔNIRS ≥ 20.9% had a significantly higher stroke rate (14.3% vs. 5.7%, *p* = 0.045) than those below the cutoff, while mortality and MACE rates were comparable (Table 4). In the multivariable-adjusted forest plot, ΔNIRS was significantly associated with risk in the overall cohort (OR = 1.04, 95% CI: 1.01–1.07, *p* = 0.022) and in the 20–24 °C subgroup (OR = 1.04, 95% CI: 1.00–1.08, *p* = 0.026; Figure 4C).

## 4. Discussion

This study retrospectively analyzed a cohort study of 619 patients undergoing aTAAD surgery with varied aortic arch repair techniques. By integrating correlation analysis, nonlinear modeling, and risk-threshold identification, we aimed to refine intraoperative cerebral protection strategies, with a particular emphasis on temperature management based on ΔNIRS.

Our main findings are as follows. Firstly, ΔNIRS demonstrated a significant and positive correlation with lower nasopharyngeal temperatures in the Total-Arch and Arch-Stent groups, suggesting that greater cerebral desaturation occurs with deeper hypothermia during complex repairs [22]. Second, we identified a predominantly positive and accelerating nonlinear relationship between ΔNIRS and the log odds of MACE in the Hemi-Arch and Total-Arch groups, indicating escalating risk with greater desaturation. A distinct pattern was observed in the Arch-Stent group, where risk peaked and then slightly declined at very high ΔNIRS values. Third, through piecewise logistic regression, group-specific ΔNIRS risk thresholds were established (8.5%, 19.6%, and 20.9%, respectively), which effectively stratified patients into distinct risk categories for adverse outcomes.

### 4.1. Interpretation of Findings and Mechanistic Considerations

The strong correlation between lower temperature and higher ΔNIRS in complex arch procedures (Total-Arch, Arch-Stent) may reflect a multifactorial physiological state [23]. Deeper hypothermia, while reducing cerebral metabolic rate, also significantly alters blood viscosity, microvascular rheology, and potentially impairs cerebral autoregulation [24]. In the context of prolonged circulatory arrest or challenging arch reconstruction, these changes may paradoxically contribute to heterogeneous microcirculatory flow or suboptimal oxygen delivery [25], captured as a larger decline in NIRS-derived regional saturation. In the Hemi-Arch group, the weaker non-significant correlation likely aligns with its shorter, less complex arrest period.

Elevated ΔNIRS is associated with increased risk of MACE. A significant decline in rSO_2_ signifies an imbalance where cerebral oxygen delivery fails to meet metabolic demands, potentially leading to cellular hypoxia [26]. This can trigger ischemic cascades, endothelial dysfunction, and neuroinflammation, culminating in clinical neurological injury or contributing to multi-organ dysfunction [27]. The GAM results suggest that the tolerance of brain for desaturation may have a nonlinear threshold [28]. Once this limit is exceeded, the compensation mechanism will fail, and the risk of injury will sharply increase. The unique risk pattern in the Arch-Stent group may suggest a selection bias where patients surviving extremely high ΔNIRS values. It may indicate the limitations of NIRS in capturing true injury burden in this specific procedural context, potentially influenced by the relatively short duration of DHCA in the Arch-Stent group.

### 4.2. Clinical Implications and the Proposed Decision Framework

The derivation of procedure-specific ΔNIRS thresholds (8.5%, 19.6%, 20.9%) moves beyond a universal alarm value and towards personalized vigilance. These thresholds, validated against hard clinical endpoints, provide tangible targets for intraoperative intervention. For instance, in a Hemi-Arch procedure, a ΔNIRS exceeding 8.5% should prompt immediate reevaluation of perfusion strategy or hemodynamic management, even during a relatively short arrest. This is corroborated by our forest plot analysis, which confirmed ΔNIRS as an independent risk factor within specific temperature ranges, most consistently in the widely used 20–24 °C zone [29].

### 4.3. Strengths and Limitations

The strengths of this study include its origin in a regional aortic center, which provides a substantial surgical volume and favorable procedural homogeneity. We employed a multimodal analytical approach, encompassing correlation analysis, nonlinear modeling, threshold detection, and adjusted multivariable analysis, enabling a more comprehensive assessment of the ΔNIRS—outcome relationship.

Several limitations must also be acknowledged. Firstly, it is a retrospective, single-center design. Second, NIRS has intrinsic limitations in clinical application; it monitors regional frontal oxygenation, which may not fully reflect global or posterior cerebral circulation, and its values can be affected by extracranial blood flow and patient-specific anatomical factors. Third, while the identified ΔNIRS thresholds are data-driven, they require external validation in independent, prospective cohorts before broad clinical adoption can be recommended.

## 5. Conclusions

In conclusion, this study demonstrates that ΔNIRS serves as a significant intraoperative monitoring indicator in aTAAD surgery, with its association to postoperative adverse outcomes being both nonlinear and dependent on the specific surgical approach. We proposed specific risk thresholds and provided preliminary supporting data, which strengthens the theoretical rationale for implementing real-time, NIRS-guided decision-making in clinical practice.

## Figures and Tables

**Table 1:** The basic data, surgical information, and prognosis in the Hemi-Arch, Total-Arch and Arch-Stent groups.

	Hemi-Arch (*n* = 127)	Total-Arch (*n* = 244)	Arch-Stent (*n* = 248)	*p*-Value
Age, years, medium	58 [22, 87]	51 [22, 83]	56 [27, 85]	**<0.001**
Gender, Male, n	91 (71.7%)	196 (80.3%)	179 (72.2%)	0.064
BMI (kg/m^2^)	24.5 [15.6, 39.8]	26.0 [16.3, 42.4]	25.8 [18.4, 40.6]	0.064
Hypertension, n	90 (70.9%)	190 (77.9%)	191 (77.0%)	0.295
Stroke history, n	10 (7.9%)	5 (2.0%)	9 (3.6%)	**0.022**
Smoke, n	48 (37.8%)	81 (33.2%)	57 (23.0%)	**0.005**
Alcohol, n	28 (22.0%)	58 (23.6%)	46 (18.5%)	0.360
*Data during the operation*				
Cerebral perfusion				**<0.001**
DHCA, n	64 (50.4%)	21 (8.6%)	82 (33.1%)	
DHCA + ACP, n	53 (41.7%)	215 (88.1%)	133 (53.6%)	
DHCA + RCP, n	10 (7.9%)	8 (3.3%)	33 (13.3%)	
Circulatory arrest temperature				0.251
<20 °C, n	29 (22.8%)	56 (23.0%)	36 (14.5%)	
20–24 °C, n	76 (59.8%)	168 (68.9%)	199 (80.2%)	
≥24 °C, n	22 (17.3%)	20 (8.2%)	13 (5.2%)	
Operation time, h	6.5 [3.0–11.0]	7.8 [3.7–16.0]	6.5 [3.7–12.5]	**<0.001**
CPB, min	204.0 [177.5, 225.0]	230.0 [185.8, 273.5]	189.0 [164.0, 220.5]	**<0.001**
Clamping time, min	155.0 [128.0, 172.0]	170.0 [128.8, 206.0]	135.0 [114.8, 161.2]	**<0.001**
Circulatory arrest time, min	25.0 [18.5, 34.0]	38.0 [31.0, 45.0]	22.0 [18.0, 28.0]	**<0.001**
NRIS baseline (dual), %	121.0 [116.8, 128.5]	125.0 [118.0, 132.0]	123.0 [118.0, 131.0]	0.1871
*Postoperative data*				
Postoperative Stroke, n	8 (6.3%)	15 (6.1%)	19 (7.7%)	0.650
In-hospital mortality, n	15 (11.8%)	23 (9.4%)	22 (8.9%)	0.777

ACP, antegrade cerebral perfusion; BMI, body mass index; CPB, cardiopulmonary bypass; DHCA, Deep Hypothermic Circulatory Arrest; RCP, retrograde cerebral perfusion. *p* < 0.05 is considered statistically significant and is displayed in bold.

**Table 2:** Comparison of demographic and clinical characteristics stratified by ΔNIRS threshold in Hemi-Arch group.

Characteristic	ΔNIRS Group (%)		*p* Value
	<8.5 (*n* = 50)	≥8.5 (*n* = 77)	
DHCA, Min, Mean ± SD	27.3 ± 11.6	25.3 ± 8.5	0.311 ^a^
Mortality, n (%)	2 (4.0%)	13 (16.9%)	**0.028 ^b^**
Stroke, n (%)	0	8 (10.4%)	**0.022 ^c^**
MACE, n (%)	2 (4.0%)	18 (23.4%)	**0.003 ^b^**

^a^ Welch Two Sample *t*-test; ^b^ Pearson’s Chi-squared test; ^c^ Fisher’s exact test. DHCA, Deep Hypothermic Circulatory Arrest; MACE, Major Adverse Cardiovascular Events. *p* < 0.05 is considered statistically significant and is displayed in bold.

**Table 3:** Comparison of demographic and clinical characteristics stratified by ΔNIRS threshold in Total-Arch group.

Characteristic	ΔNIRS Group (%)		*p* Value
	<19.6 (*n* = 205)	≥19.6 (*n* = 39)	
DHCA, Min, Mean ± SD	38.7 ± 9.6	35.8 ± 12.4	0.184 ^a^
Mortality, n (%)	16 (7.8%)	7 (17.9%)	0.068 ^c^
Stroke, n (%)	8 (3.9%)	7 (17.9%)	**0.004 ^c^**
MACE, n (%)	23 (11.2%)	9 (23.1%)	**0.044 ^b^**

^a^ Welch Two Sample *t*-test; ^b^ Pearson’s Chi-squared test; ^c^ Fisher’s exact test. DHCA, Deep Hypothermic Circulatory Arrest; MACE, Major Adverse Cardiovascular Events. *p* < 0.05 is considered statistically significant and is displayed in bold.

**Table 4:** Comparison of demographic and clinical characteristics stratified by ΔNIRS threshold in Arch-Stent group.

Characteristic	ΔNIRS Group (%)		*p* Value
	**<20.9 (*n* = 192)**	**≥20.9 (*n* = 56)**	
DHCA, Min, Mean ± SD	24.1 ± 8.2	25.9 ± 16.4	0.442 ^a^
Mortality, n (%)	15 (7.8%)	7 (12.5%)	0.289 ^c^
Stroke, n (%)	11 (5.7%)	8 (14.3%)	**0.045 ^c^**
MACE, n (%)	25 (13.0%)	13 (23.2%)	0.062 ^b^

^a^ Welch Two Sample *t*-test; ^b^ Pearson’s Chi-squared test; ^c^ Fisher’s exact test. DHCA, Deep Hypothermic Circulatory Arrest; MACE, Major Adverse Cardiovascular Events. *p* < 0.05 is considered statistically significant and is displayed in bold.

## Data Availability

The data presented in this study are available on request from the corresponding author.

## Supplementary Materials

The following supporting information can be downloaded at: https://www.mdpi.com/article/10.3390/jcdd13010012/s1, Figure S1: The piecewise logistic regression model in three groups (A = Hemi-Arch Group; B = Total-Arch Group; C = Arch-Stent Group); Table S1: The additional basic data and surgical information; Tables S2–S4:Threshold effect analysis of ΔNIRS on MACE in Hemi-Arch Group(S2), Total-Arch Group(S3) and Arch-Stent Group(S4). File S1: Methods of Surgical procedures.

## Abbreviations

ACP	Antegrade cerebral perfusion
ANOVA	Analysis of Variance
aTAAD	Acute Stanford type A aortic dissection
BCP	Bilateral cerebral perfusion
CICU	Cardiothoracic intensive care unit
DHCA	Deep hypothermic circulatory arrest
ICU	Intensive care unit
NIRS	Near-infrared spectroscopy
RCP	Retrograde cerebral perfusion
TTE	Transthoracic echocardiography
