# Supplementary material for: Perioperative Cerebral Protection and Monitoring of Acute Stanford Type A Aortic Dissection: A Retrospective Cohort Study
Source: J Cardiovasc Dev Dis. 2025 Dec 24;13(1):12. doi: 10.3390/jcdd13010012 (PMC12841893; doi:10.3390/jcdd13010012)
Supplement: Supplementary file 1 [file jcdd-13-00012-s001.zip › Supplementary File S1.pdf]

Supplementary File S1

Methods

Surgical procedures

All patients underwent combined intravenous and inhalation anesthesia and routine tracheal intubation. Arterial blood pressure was monitored by artery puncture in the upper and lower extremities during the operation, and esophageal ultrasound probes were routinely placed (except for contraindications such as esophageal lesions).

All open surgical methods were median sternal incision. Prior to routine thoracotomy, the right axillary artery and/or femoral artery were exposed according to dissection involvement, intubation selection, and cerebral perfusion. After the thoracotomy was performed through a median sternal incision, the supraricular branch vessels were exposed and the tape was used as a reserve. After systemic heparinization (300IU/kg), we established cardiopulmonary bypass based on selected arterial catheterization combined with right atrial catheterization or superior and inferior vena cava catheterization. After occlusion of the aorta, blood arresting fluid was injected at the root of the aorta or through the coronary ostium. Myocardial arresting fluid was injected forward through the coronary ostium or reverse through the coronary sinus every 15-20 minutes during the operation. All surgeons will decide the surgical method according to the scope of dissection.

After completing the distal bow operation, the core temperature is restored. During the rewarming phase, we treat aortic root lesions. Aortic valve replacement is required only for patients with severe aortic valve damage. The indications for root replacement (Bentall or David surgery) during the same period are root dilation (≥45mm), root intimal laceration, and

severe coronary artery involvement. In addition, most patients can be treated with root lesions through root repair surgery (for specific methods, see the aforementioned literature report).
