# Supplementary figures and images for: Perioperative Cerebral Protection and Monitoring of Acute Stanford Type A Aortic Dissection: A Retrospective Cohort Study
Source: J Cardiovasc Dev Dis. 2025 Dec 24;13(1):12. doi: 10.3390/jcdd13010012 (PMC12841893; doi:10.3390/jcdd13010012)

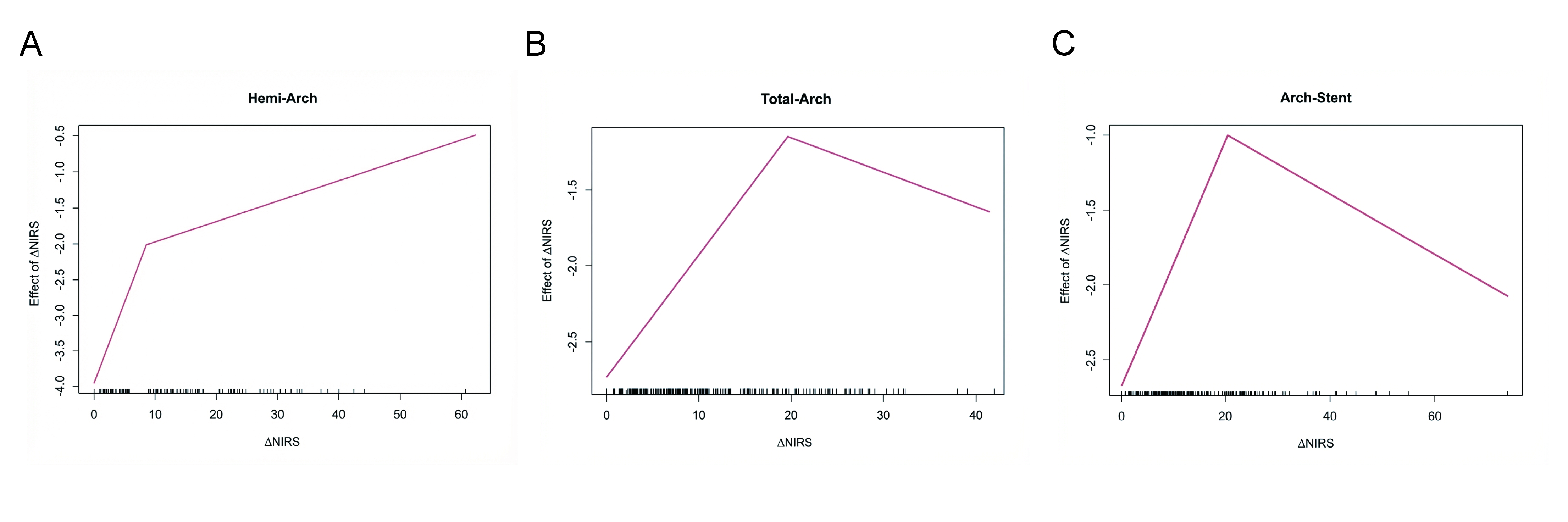

Supplement: Supplementary file 1 [file jcdd-13-00012-s001.zip › Sup Figure S1 piecewise.tif]
